# Crop diversity and stability of revenue on farms in Central Europe: An analysis of big data from a comprehensive agricultural census in Bavaria

**DOI:** 10.1371/journal.pone.0207454

**Published:** 2018-11-19

**Authors:** Robert Weigel, Thomas Koellner, Patrick Poppenborg, Christina Bogner

**Affiliations:** 1 Professorship of Ecological Services, BayCEER, University of Bayreuth, Bayreuth, Germany; 2 Ecological Modelling, BayCEER, University of Bayreuth, Bayreuth, Germany; 3 Experimental Plant Ecology, Institute of Botany and Landscape Ecology, University of Greifswald, Greifswald, Germany; Fred Hutchinson Cancer Research Center, UNITED STATES

## Abstract

Diversity of agricultural landscapes is important to maintain the provision of ecosystem services. In face of decreasing support measures for agricultural markets in the European Union, diversified crop portfolios could also offer a possibility to stabilize revenue at farm level (portfolio effect). We hypothesize that (i) diversity of crop portfolios changes along spatial gradients in the study area (Bavaria, Germany), (ii) the composition of portfolios depends on farm parameters, and (iii) more diverse portfolios on arable land provide higher revenue stability. We analysed agricultural census data comprising all farms (*N* = 105 314) in the study area and identified 26 typical crop portfolios. We show that portfolio composition is related to farm characteristics (whole farm revenue, farm type, farm size) and location. Currently, diversification of crop portfolios fails to promote stability of portfolio revenue in the study area, where policy still indirectly influences market prices of energy crops. We conclude that the portfolio effect as a natural insurance was less important in recent years due to high market prices for specific crops. This low need for natural insurances probably favoured simplified portfolios leading to decreased agricultural diversity.

## Introduction

Crop diversity is an important part of agrobiodiversity and is related to ecosystem services provided by agroecosystems [1]. For example, crop diversity was reported to sustain soil quality, to buffer yield variance against adverse weather events and to substitute fertilizer use while maintaining economic competitiveness [2–4]. Moreover, crop diversity can increase wildlife habitat quality by reducing the use of agrochemicals [5]. For a farmer to maintain ecosystem services, crop diversity can be realized across time on the field scale by crop rotation on one hand [6, 7]. On the other hand, diversifying the portfolio of cultivated crops maintains diversity in space on the farm scale. On both temporal and spatial scales, provisioning, supporting and regulating ecosystem services benefit from diversified farming [4, 5, 8].

On the spatial scale, a diverse crop portfolio provides an insurance effect to mitigate revenue variability at farm level [9–11]. This insurance effect is based on the portfolio theory stating that an investor can reduce financial risk by diversifying the portfolio of assets [12]. In the European Union (EU) financial risks were buffered by market support measures (before 1990) and by direct payments coupled to production, favouring agricultural intensification and specialization [13–15]. So, portfolio composition at farm level was strongly influenced by those market support measures in the EU agricultural market. Taking a step towards less regulated markets, the large share of coupled direct payments were replaced in stages by decoupled direct payments between 2005 and 2013 [14]. In this time of less regulated markets, risk awareness of farmers should increase [9, 10, 16]. Following portfolio theory, crop portfolios could then be diversified to scatter income risk as much as possible [9–11]. The diversification and risk scattering may be a natural income insurance that could substitute reliance on financial insurances against yield failure [17]. In turn, availability of financial insurances and regulated markets may decrease the need for this natural income insurance and favour simplified portfolios [17]. In consequence, policy should aim at stimulating farmers to diversify their portfolios as a natural income insurance because the public would also profit from the increased agrobiodiversity and resulting provision of ecosystem services [10]. Hence, it has to be critically analysed, if this spatial diversification really offers this insurance effect and if less regulated markets consequently really lead to higher crop diversity. For this critical analysis within a certain political environment, the relationship between revenue stability and portfolio diversity has to be understood.

Besides rationally composing portfolios to adjust for market risks, personal preferences of the farm holder influence portfolio composition [13, 18]. The choice is further constrained by the physical and socio-economic environment in which a farm is situated [4, 19]. In the physical landscape, climate conditions and soil quality have a strong impact on crop choice if aiming at the optimization of yield under the respective local conditions [19, 20]. Beyond this, portfolio composition is influenced by farm specific parameters. Bradshaw [5] reported that portfolio diversity is higher on larger farms. This might be related to the fact that small farm holders are in greater need to complement their low farm household revenue with income from off-farm work. This off-farm income makes them more independent from on-farm yield and revenue risk [21]. Correspondingly, on those part time farms there is less need for low-risk portfolios which could result in a higher input of agro-chemicals as well as in a decreased portfolio diversity compared to larger farms [13, 21, 22].

Direct political guidelines also influence farmers’ decision-making. In particular in organic framing, the Common Agricultural Policy (CAP) of the EU demands increased portfolio diversity [23]. Furthermore, organic farming restricts input of industrial fertilizer, so that portfolios have to be more diverse to compensate for the missing external input [24]. Accordingly, more diverse portfolios could be found in organic farming. Furthermore, the degree of specialization influences portfolio diversity. Specialized farms rely on high external chemical input that goes along with simplified portfolios, while integrated farms (crop and livestock) are more related to decreased external input and more circular economy so that more diversified portfolios are realized [25]. If, however, a farm might be diversified in manifold ways by involvement in permanent crop cultivation, animal husbandry, tourism, and off-farm work, this leaves less time and need for diversification of crop portfolios on the arable land of the farm. Thus, farms with a high income complementing arable land use might have low portfolio diversity on arable land [21, 26]. Besides diversification of the crop portfolio, crop rotation systems can be used to balance productivity on the field scale [6]. Relying on crop rotation systems could thus be another factor that decreases the need to diversify crop portfolio on farm level.

Since crop diversity and portfolio composition are essential for ecosystem services in the agricultural landscape, it is important to analyse how the physical and socio-economic environments constrain the portfolio composition. Only thus can we understand how policy could enhance portfolio diversity and how clear policy schemes for enhancing ecosystem services in agricultural systems could be developed. To our knowledge, most studies that tried to find the connection between crop portfolio composition and ecological, economic and farm-specific factors concentrated on a small number of sample farms. Given the multitude of interactions, however, this link might be better understood from a larger geographic perspective—an approach we pursue in this work.

Our goal was to analyse regionally specific crop portfolios and to give further insights into how the choice of crops and composition of crop portfolios is influenced by geographical and socio-economic farm parameters in Bavaria (Germany). In an approach combining both descriptive and correlative techniques for analysis of large data sets, all farms (*N* = 105 314) of the complete Free State of Bavaria were incorporated in one analysis. We determine typical crop portfolios for farms in Bavaria and analyse their diversity on the spatial scale. We further evaluate the study-area specific relationship between portfolio diversity on arable land and temporal revenue stability of those portfolios. Since the whole analysis is based on the diversity of these farm-specific crop portfolios, the work is clearly focussed on the farm scale. We test the hypothesis that portfolio diversity changes along spatial gradients (H1). This is based on the assumption that agriculture is adequately adapted to local geographic (physical and economic) conditions. Moreover, we hypothesize that portfolio composition of individual farms depends on socio-economic parameters and that higher diversity is found on larger farms, in full-time compared to part-time farming, on farms integrated arable farming and animal husbandry, in farming without intra-annual crop rotation, and on farms where whole farm revenue largely originates from arable land use (H2). Testing the portfolio theory in Bavarian agriculture, we hypothesize that more diverse portfolios offer a more stable risk–revenue relationship (H3).

## Materials and methods

### Study area

**Fig 1:**
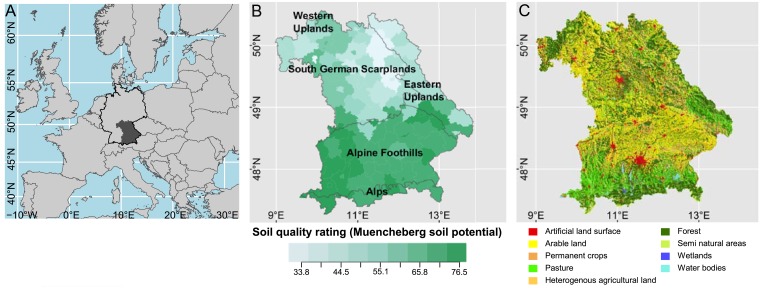
Study area Bavaria in Germany. (a) location (https://cran.r-project.org/web/packages/rworldxtra/index.html, accessed on 2018-01-09, derived from Natural Earth data), (b) large landscapes and soil quality rating (yield potential, full range going from 0 (low) to 100 (high)). Data by Bundesanstalt für Geowissenschaften und Rohstoffe (Federal Institute for Geosciences and Natural Resources) (data source: SQR1000 v1.0, BGR, Hannover, https://www.bgr.bund.de/DE/Themen/Boden/Ressourcenbewertung/Ertragspotential/Ertragspotential_node.html, accessed on 2017-12-16) and Bayerisches Landesamt für Umwelt (Bavarian Environment Agency) (www.lfu.bayern.de/natur/naturraeume, accessed on 2017-12-16) incorporating data by Geodaten—Bayerische Vermessungsverwaltung (Bavarian Surveying and Mapping Authority), and (c) land cover derived from Corine classification, basic year 2006. Data by the European Environment Agency (https://www.eea.europa.eu/data-and-maps/data/clc-2006-raster-4, accessed on 2017-12-16).

Our study area is the Free State of Bavaria, Germany (Fig 1A). Due to differences in geology and climate (S1 Fig), soil quality in terms of agricultural potential (Muencheberg soil potential [27]) decreases from south to north (Fig 1B). Of Bavaria’s total area (70 553 km^2^) almost half is used for agriculture and one third for forestry [28]. Two thirds of the agricultural land is managed as arable land, mostly on areas with higher agricultural potential (Fig 1C). More than half of the arable land is used to grow cereals, the remaining part for (in declining order) fodder crops, commercial crops, root crops, fallow land, and gardening. The typical farm size in Bavaria equals 10 to 50 ha (S1 Table).

### Data description

For the analysis of land use and socio-economic variables of Bavarian farms an agro-economical census of Bavaria, the *Landwirtschaftszählung* (lz2010) was available. lz2010 is part of a coordinated agricultural census conducted in the year 2010 across all 27 EU member states [29]. The data was made available by the Bavarian Statistical Office (LfStat: *Bayerisches Landesamt für Statistik*). In Bavaria, the census included all officially recorded farms (*N* = 105 314). Since we focussed our study on analysing arable land use only, we excluded forestry and agricultural businesses without any arable land (mostly enterprises specialized in animal farming only). In doing so, a total of *N* = 79 532 farms remained that correspond to the total area of arable land in Bavaria corresponding to one third of the state area.

Land use on each farm was characterized by the area share in percent of 41 crop categories of either single crops or crop families (e.g. *vegetables and strawberries* listed as one crop category; S2 Table). General data about the farms in the year 2010 contained information about location, annual whole farm revenue (estimated total agricultural production without costs), type of farming, soil cultivation (area share of applied method in percent) and size of arable land (S3 Table). Individual costs are not part of the census due to data privacy. We refrained from estimating the mean farm costs because they are not representative for the individual way of conducting farm business (e.g. intensity of using resources).

A high level of data protection had to be respected when analysing the census data. Thus, no minimum and maximum values are displayed in graphics referring to lz2010 census data. This is the case because the minimum and maximum values refer to specific farms (e.g. the smallest and the largest farm in Bavaria). Displaying these values would theoretically enable a re-identification of specific farm holders and their confidential personal data.

Time series of yield for the most frequent crops in Bavaria are publicly available at the Bavarian *GENESIS* database (www.statistikdaten.bayern.de, accessed on 2017-10-17) provided by the LfStat and access to data not listed in the database was granted by LfStat (S6 and S7 Figs). Similarly, price data of agricultural commodities is publicly available at national level in the *GENESIS* database (www-genesis.destatis.de, accessed on 2017-10-17) provided by the Federal Statistical Office (*Statistisches Bundesamt*) and by eurostat (www.ec.europa.eu/eurostat, accessed on 2017-10-17). Prices of green maize and legumes had to be calibrated and reconstructed referring to the price of grain maize. Fallow and unused land were assumed to be zero revenue commodities because we only focus on stability of farm revenue on arable land measured by immediate cash return. Just as for fallow land, we also leave considerations about long-term return from other ecosystem services aside for all other crop categories. Price data and yield data were used to calculate the annual revenue per area and crop (without costs). To calculate the portfolio revenue, the revenue of related individual crops was weighted by their area share. The revenue for the crop category *vegetables and strawberries* was calculated from the price and yield data of 1/3 asparagus, 1/3 carrots, and 1/3 strawberries. We did so because the agricultural census only reports the area used for vegetable and strawberry cultivation in summary without further details on which vegetables were cultivated at each individual farm. Given the high importance of asparagus and carrot cultivation in Bavaria [30] and with regard to availability of yield and price time series, we thus built this exemplary model portfolio composition to represent the category vegetables and strawberries.

### Empirical crop portfolios

A set of representative crop portfolios for Bavaria were empirically derived from the lz2010 survey data by clustering according to the relative area share of crops cultivated on individual farms in the survey year 2010. This grouping approach was based on the Clustering of Large Applications algorithm (clara). As described by Kaufmann and Rousseeuw [31], clara draws *S* sub-samples of size *n* = *s*. Each is clustered around *k* medoids (geometrical centres). Subsequently, the whole data set is clustered around the *k* medoids of the optimal sub-sample determined by the lowest mean distance between points in each cluster. The number of clusters *k* was determined by maximizing the overall average silhouette width. It compares the similarity of points inside their assigned cluster to the similarity to all other clusters and is a measure of the quality of clustering. We optimized the number of clusters in two steps [32]. After a coarse optimization (*k* = 2, …, 65, *S* = 250, *s* = 500) *k* was fine-tuned (*k* = 20, …, 30, *S* = 100, *s* = 1000), which yielded an optimal *k* = 26. Although the optimum was not really pronounced, *k* = 26 represents a manageable number of clusters (S2 Fig).

The whole data set was finally clustered by clara with *k* = 26, *S* = 100, and *s* = 2000. Thus, each farm was assigned to one of the 26 empirical portfolios in Bavaria. The crops contributing cumulatively to ≥ 50% of the area of each portfolio determined its label referred to later in the article.

Diversity of portfolios was quantified by Shannon–Wiener Diversity Index (*H*′). The index refers to the sum of log-scaled shares of (crop) categories. Thus, crop number and evenness of area shares (abundance) is accounted for. Mentioning of *diversity of portfolios* in this manuscript always refers to diversity according to Shannon–Wiener diversity index.

### Explaining portfolio composition

In a classification with random forest [33] we evaluated how well farm characteristics could explain the composition of portfolios. Note that we did not use this information to cluster the data and generate the portfolios. In a random forest model *n*_*tree*_ decision trees are grown based on *n*_*tree*_ bootstrapped sub-samples. The trees are decorrelated considering only a random subset *m* of all predictors *p* at each node (in this study chosen as $m\approx \sqrt{p}$ [33]). The *out of bag error* (the prediction error for classifying each data sub-set that was not part of the *n*_*tree*_ bootstrapped sub-samples) and the *mean decrease in accuracy* (the percentage loss of accuracy when removing a certain variable from the model) were used as a measure of prediction accuracy (for each class and for the whole model) and measure of importance of predictors, respectively.

We built the random forest model to explain how the composition of empirical portfolios was related to different farm characteristics, namely acreage (size of arable land), economic type (status of off-farm work), farm type, status of ecological farming, status of inter cropping (the census summarized over the practices of relay inter cropping and cultivation of a catch crop between two consecutive main crops), location (administrative district), and whole-farm revenue (total revenue of on-farm production) (detailed overview of levels of categorical variables is given in S3 Table). The whole-farm revenue was included in the model as the whole-farm revenue per farm and we also calculated the share of the whole-farm revenue per hectare of arable land. This was taken as a measure for on-farm diversification besides arable farming (high whole-farm revenue per hectare indicates higher revenue generated from other sources than arable land use). Moreover, whole farm revenue may also depend on local environmental conditions, so that these are also taken into account indirectly. We used the optimal clara sub-sample as training set. For each tree in the random forest regression, stratified bootstrap samples were drawn, so that the target variable ‘portfolio’ was equally represented (S4 Table). The parameter *n*_*tree*_ was chosen large enough to ensure good model performance (S3 Fig) [34, 35]. The quality of prediction was tested against the remainder of the data by calculating the classificaton error for each portfolio class and for the whole model.

### Stability analysis

From the revenue of each portfolio we calculated the trend free standard deviation (SD) of portfolio revenue according to the previous use of SD or variance in agronomy to estimate yield variability [36] and revenue volatility [4, 37]. Since portfolio revenue was not free of trends, SD was calculated after subtracting a local first order polynomial smoothing spline from the original time series (S7 Fig). We calculated SD for the years 2000–2013. We assumed that our empirical portfolios of the year 2010 are representative for this period between two major CAP reforms (‘Agenda 2000’ in 1999 and the new CAP reform in 2014).

We used a high SD as an indicator for high revenue volatility. We described the portfolio effect by evaluating how revenue stability was correlated with diversity of portfolios (*H*′) by calculating Spearman’s correlation coefficient *ρ*. We tested the hypothesis of no correlation (*H*_0_: *ρ* = 0) in a 1000-fold bootstrap procedure with *α* = 0.05 as significance level.

All calculations were done in R [38] using various add-on packages (S1 Appendix).

## Results

### Empirical crop portfolios

We found 26 empirical portfolios consisting of one to six crops by cluster analysis (clara). Six portfolios were dominated by maize and contained a low number of cultivated crops, from one to three (S5 Table). Eight portfolios were dominated by the grain crops wheat, barley, triticale, rye, and oats. Those grain portfolios consisted of one to five crops (only triticale forming a single-crop portfolio). In portfolios dominated by a mix of grains, maize or other crops, three to seven crops were cultivated. So the maize portfolios were poorest in crop species richness, grain crop portfolios had medium crop species richness, and grain-mixed portfolios had highest species richness.

### Spatial distribution of empirical crop portfolios and their diversity (H1)

**Fig 2:**
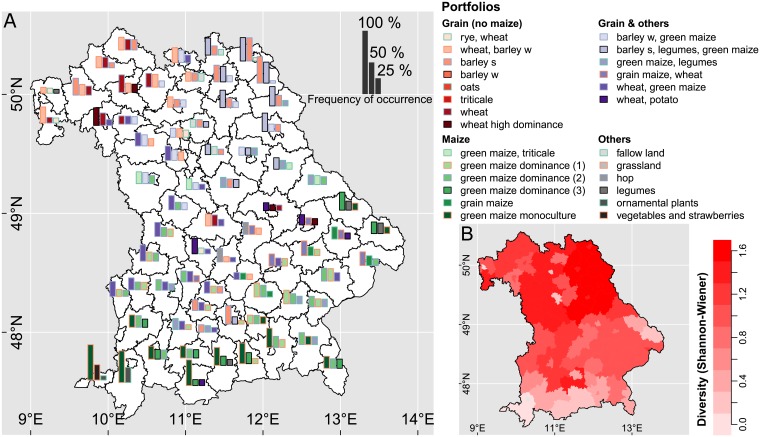
Diversity of crop portfolios in Bavaria. (a) Spatial distribution and frequency (height of bars) of the three most important empirical crop portfolios in rural districts. Labels are inherited from the most dominant crops (accounting for more than 50% of the area of each portfolio) and (b) Shannon–Wiener diversity index (mean) of the three most important empirical crop portfolios.

Species-poor, maize-dominated portfolios were typical for the South (prevalence strongly expressed) and spring barley, winter barley, and wheat (winter wheat) dominated more species-rich portfolios typical for the North (Fig 2A). Consequently, portfolio diversity (*H*′) increased from south to north (Fig 2B). In the central part of Bavaria, portfolios under at least partial dominance of wheat were more frequent. In the north-western tip of Bavaria portfolios of wheat in high dominance or wheat in combination with other grain crops were more important. The low diversity portfolio *wheat high dominance* occurred as one of the most frequent portfolios in the districts of Würzburg, Regensburg, Schweinfurt, and Straubing only. The more diverse portfolios (combinations of spring and winter barley together with green maize and legumes) were most important in north-eastern Bavaria.

Portfolios of speciality crops (fruits and vegetables, horticulture, and floriculture crops) were more frequent in the urban than in the rural districts of Bavaria. Both portfolios *ornamental plants* and *vegetables and strawberries* belonged to the most frequent portfolios in the urban districts of Bamberg, Fürth, Munich, and Nuremberg (S4 Fig).

### Farm characteristics and portfolio composition (H2)

**Fig 3:**
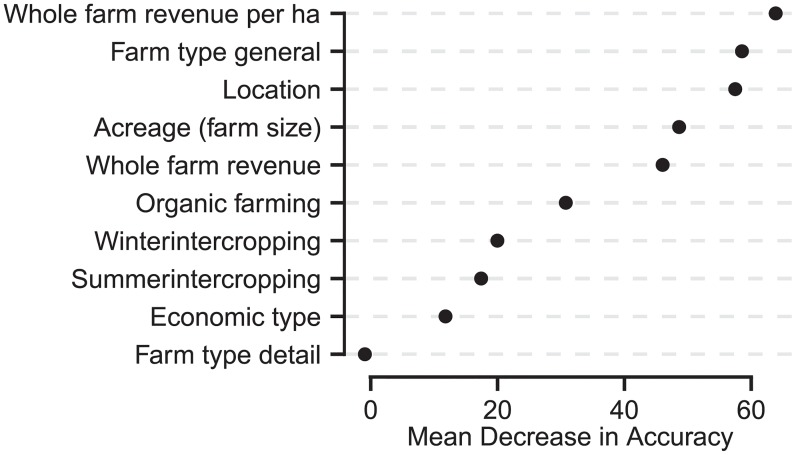
Importance of the random forest predictors explaining the relationship between the composition of empirical crop portfolios and farm characteristics. Importance is given as mean decrease in accuracy in percent showing the loss of accuracy when removing a certain variable from the model. The whole-farm revenue (per hectare) is based on the total agricultural production of each farm in the year 2010 (per hectare).

The most important socio-economic parameters linked to portfolio composition were whole-farm revenue per hectare, general farm type, and location (Fig 3). Acreage (i.e. size of arable land) and whole-farm revenue were further important predictors followed by organic farming as a less important predictor. A further strong decrease in model accuracy separated crop rotation (inter cropping) and status of part time farming (economic type) as unimportant variables. The model had an out of bag error of 72.4% (training set) and a prediction error of 74.0% (test set).

**Fig 4:**
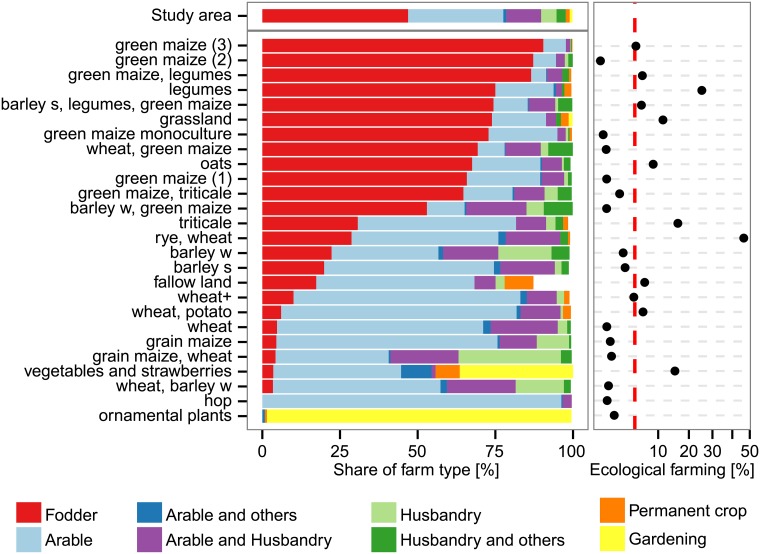
Share of general farm types and share of organic farming within empirical crop portfolios. Portfolios are labelled according to their most dominant crops (accounting for more than 50% of the area of each portfolio). Stacked bars of farm types not adding up to 100% are due to unclassified observations.

**Fig 5:**
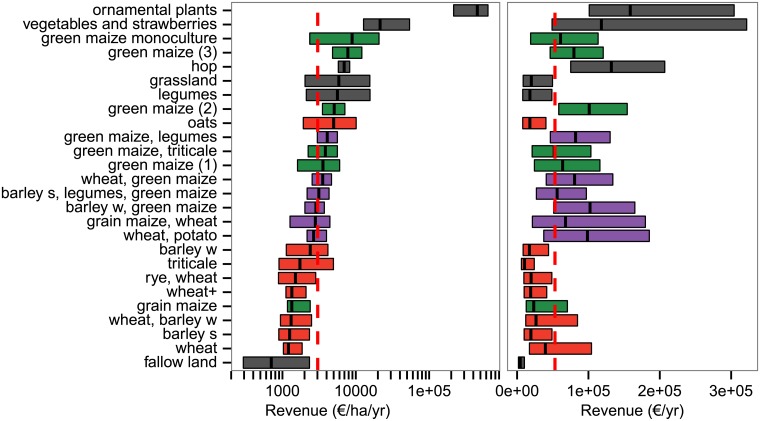
Whole-farm revenue of farms for empirical crop portfolios. Portfolios are labelled according to their most dominant crops (accounting for more than 50% of the area of each portfolio). Whole-farm revenue is given as whole-farm revenue per hectare (left) and as whole-farm revenue (right) of the year 2010. Boxes refer to the first quartile, median, and third quartile of the data. Colours refer to grain dominated portfolios (red), maize dominated portfolios (green), portfolios being dominated by grain, maize, and other crops (purple) and other portfolios (grey).

Large acreage (size of arable land) was linked to the more diverse portfolios dominated by a mixture of grain, maize and other crops (S5 Fig). Grain dominated portfolios were found on farms of various sizes. The maize portfolios, which all had a rather low diversity, were cultivated on farms with average to below average acreages. Smallest farms cultivated low diversity portfolios of speciality crops, such as the *ornamental plants* portfolio and *vegetables and strawberries* portfolio (small farms with high revenue, Figs 4 and 5). In summary, the size of arable land was an important predictor for portfolio composition; portfolios with higher diversity were cultivated on larger farms.

Highest shares of organic farming were linked to the *rye, wheat* (higher diversity), the *legumes*, the *vegetables and strawberries*, the *triticale* (one-crop portfolio, lowest diversity), and the *grassland* portfolios (Fig 4). Lower shares of organic farming were related to maize dominated portfolios (generally moderate to low diversity) as well as to portfolios of mixed dominance of grain, maize and other (non-grain) crops (generally higher diversity). Thus, although the status of organic farming was relevant for portfolio composition in general, there was no clear relationship between organic farming and diversity of portfolios.

The maize portfolios were mostly found on farms specialized on fodder production while grain-mixed portfolios were related to both, fodder cultivating farms and integrated farms (arable land use and animal husbandry) (Fig 4). Grain dominated portfolios were more related to farms specialized on arable farming. There was thus a trend from lower diversity portfolios belonging to farms specialized on fodder production (maize portfolios), over moderate diversity portfolios found in pure arable farming (grain portfolios), to higher diversity portfolios belonging to integrated farms (portfolios of mixed dominance of grain, maize, and other crops).

All green maize portfolios were grown on farms with a high whole-farm revenue per hectare. Similarly, farms growing portfolios of speciality crops also had high whole-farm revenue per hectare (Fig 5). Grain-mixed portfolios showed an average whole-farm revenue per area and grain portfolios were mostly found on farms with a low whole-farm revenue per area. We detected a strong relationship between the whole-farm revenue and portfolio composition. Portfolios with the highest diversity were related to farms with average whole-farm revenues per hectare. In addition, portfolio diversity decreased for low as well as for high revenues.

### Portfolio diversity and risk free return (H3)

**Fig 6:**
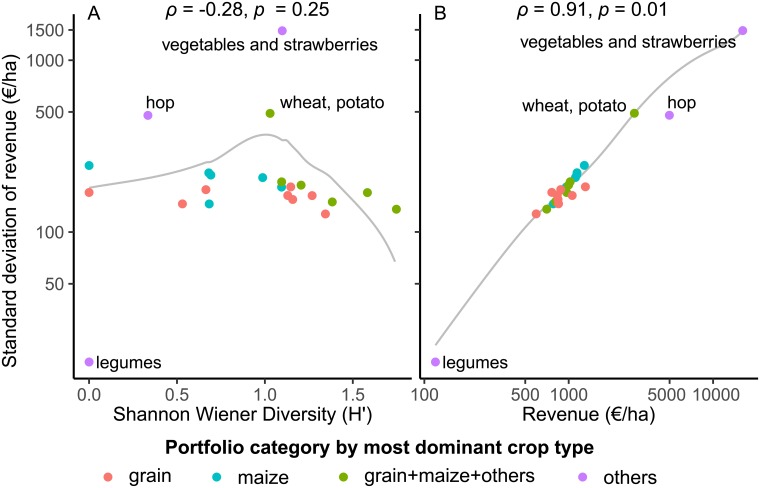
Relationship between (a) standard deviation of revenue (SD) and diversity (Shannon–Wiener Index *H*′) as well as between (b) SD and mean revenue for empirical crop portfolios. Spearman’s correlation coefficient was tested for significance (*p* ≤ 0.05) in a 1000-fold permutation test.

The diversity of empirical crop portfolios ranged from *H*′ = 0 (single crop portfolios) to *H*′ = 1.7. Maize dominated portfolios were found at the lower end of the diversity scale. Grain portfolios scattered from low to relatively high values and mixed portfolios scattered from moderate to highest values along the diversity scale (Fig 6A). Risk volatility (SD) was not correlated to diversity. The portfolios of speciality crops (*hop* and *vegetables and strawberries* portfolio) with their high revenue had exceptionally high risk volatility compared to the other portfolios, which corresponds to the strong link between risk volatility and mean revenue in general (Fig 6B; S5 Table).

## Discussion

### Diversity of portfolios increases on poorer soils (H1)

The diversity of portfolios increases from southwest to northeast. This could be explained by the soil quality and agricultural potential, which are higher in the south of the study area and in the district of Würzburg than in the rest of Bavaria (Fig 1). Modern agricultural intensification and simplification is strongly climatically constrained and prone to yield failures due to adverse weather events [39, 40]. Higher soil quality can buffer the effect of these adverse weather events on crop performance more efficiently [4]. Thus, on soils with higher quality, agriculture can be intensified towards simplified portfolios to increase the income with less concern for risk of yield failure [4, 19]. Furthermore, the regions of the study area with poorer agricultural or economical potential receive a compensatory allowance (*Ausgleichszulage*) from the EU, the German state, and the federal state of Bavaria [41]. The compensatory allowance is granted if specialization on intensified cultures (maize, wheat, sugar beet, hop, and vegetables) is avoided. As a result, both poorer soil quality and compensatory allowances increase crop diversity of portfolios in areas of Bavaria with lower agricultural potential. Accordingly, location (administrative district) was a strong predictor in the random forest model for portfolio composition. In addition, we could show a clear gradient of portfolio diversity increasing from south to north. In agreement with our hypothesis, this highlights that due to physical and political conditions farm location is an important constraint for portfolio composition for Bavarian farmers.

### Portfolio composition depends on socio-economic characteristics of individual farms (H2)

We could confirm that more diverse portfolios were cultivated on larger farms. This is partly due to Common Agricultural Policy in the EU, which demands a minimum of two or three crops for farms larger than 10 or 30 hectares [14, 23]. Furthermore, integrated farming (arable land use and animal husbandry) that was connected to more diverse portfolios, was practised on larger farms. Fodder production for farm livestock on those large, integrated farms requires cultivation of a variety of forage and protein rich grain crops. This results eventually in planting diversified portfolios of maize and grain crops complemented by legumes and oil seeds [42]. Thus, a diversified crop portfolio on integrated farms may be seen as consequence of animal husbandry on one hand. On the other hand, the integration of animal husbandry with diverse crop portfolios may also directly arise from a farmer’s decision to optimally balance farm productivity and supporting ecosystem services (e.g. nutrient recycling) in closed-loop farming [13, 43, 44].

Our results show that diversity of crop portfolios is lower on smaller farms. As previously discussed, our results also show that farmers within the same region tend to choose the same portfolios. Thus, crop diversity at the regional scale may profit from larger, integrated farms. On the local scale, however, Fahrig et al. [45] showed that agrobiodiversity and ecosystem services benefit from smaller field size. Consequently, policy could aim at reducing the field size on large integrated farms to promote both agrobiodiversity on the local scale and crop diversity on the landscape scale. Alternatively, Prager [46] suggested that agri-environmental schemes should be increasingly adjusted across collaborations of farms, to relieve small farms in particular from the demand to individually diversify the landscape. This is already partly recognized in CAP and proved to be a valuable tool to maintain ecosystem services in agriculture on the landscape scale [46].

The farm type ‘organic farming’ was a less important predictor for portfolio composition. This weak relationship between organic farming and portfolio composition might contradict the assumption that organic farming implies different decision making than conventional farming [47, 48]. However, the weakness of the relationship could be an artefact of the random forest analysis. In fact, random forest prefers continuous predictors and those with many categories [49] and the effect of organic farming on portfolio composition might have been underestimated. Organic farming was related to very low to very high diversity portfolios. This contrasts the hypothesis of organic farming being more diverse [50]. On the one hand, EU regulations for organic farming demand increased agrobiodiversity [23]. On the other hand, the income security supplied by subsidies for organic farming could also encourage farmers to maximize short-term income with more risky, simplified portfolios [50]. Thus, our results showed that organic farming indeed affected the portfolio composition, however, not in a simple and linear way.

The whole-farm revenue was an important predictor for portfolio composition. The portfolios with highest diversity (mixed dominance of grain, maize, and others) were related to farms with average whole-farm revenue per hectare. As previously discussed, those portfolios are strongly related to integrated farms and diversity is a by-product of supplying the livestock with fodder. Additionally to arable farming, those farms obviously complement their revenue with animal husbandry. In contrast, those farms that rely on arable land use only have a lower whole-farm revenue per hectare. Thus, the moderately diverse grain dominated portfolios were found on farms with below average whole-farm revenue per hectare because those portfolios are typical for farms specialized on arable farming. The low diversity portfolios dominated by maize or portfolios of speciality crops were found on farms with high whole-farm revenue per hectare. As was pointed out earlier, the maize portfolios were strongly linked to the farm type specialized on fodder production. By definition of the lz2010 survey, this farm type also includes animal husbandry [29]. Therefore, in case of maize portfolios, this high whole-farm revenue per hectare might also be related to farms complementing their budget with animal husbandry. Here, arable land use can be simplified because revenue volatility from arable land use can be financially compensated by other farm activities [21, 26]. In contrast, speciality crops offer high revenue from arable land (high portfolio revenue), so that high whole-farm revenue is also typical for highly specialized farms. We found that the whole-farm revenue is strongly related to portfolio composition and portfolio diversity. Following the above argumentation, however, the whole-farm revenue is closely related to farm type in the study area. So farm type could actually be related to portfolio composition, while the relationship to the whole-farm revenue could be an indirect correlation.

In summary, portfolio composition was related to farm characteristics and location. Our findings highlight that additionally to the constraints of the physical and political environments, farm characteristics restrict the set of portfolios, from which equally constrained farmers could choose. With these strong local differences and impact of socio-economic situation of a farm, policy schemes that aim at maintaining diversity and ecosystem services are of more use if adjusted to the regional and farm situation [51].

### Stability of income does not increase with portfolio diversity (H3)

Farmers’ adaptation to short-term market situations and optimization of revenue sometimes favours intensified farming and selection of simplified rather than diversified portfolios [5]. Moreover, choosing simplified portfolios to achieve a higher mean revenue may generally counterbalance taking higher risks [37]. This is also suggested by our results. Contrasting our hypothesis, stability of revenue did not increase with portfolio diversity in the study period 2000–2013. However, the risk to revenue ratio was very much the same for all portfolios in our study area, which is in line with findings of Abson et al. Abson2013 for the United Kingdom. In Germany, the demand for green maize and rapeseed as energy crops is currently high and agricultural landscapes are increasingly characterized by cultivation of rapeseed or maize in monoculture [3]. While in former times cultivation of potatoes was wide spread in the study area, it is now of minor importance in the portfolios and reflects the recent trend of decreasing potato cultivation in Germany [52]. As such, the present decline in potato cultivation is an example for consequences of economic rationalism in face of declining market potential for certain crops [52]. Increasing global demand for major energy and food crops (wheat, maize, rapeseed) makes it more economically rational to simplify portfolios aiming at short-term profit [3, 4, 53]. The revenue peaks that can be achieved with those energy crops overrule revenue variability. Especially in South Germany, both increasing biogas production that was promoted by the German Renewable Energy Act (*Erneuerbare Energien Gesetz, EEG*) and increasing livestock density additionally favoured such market situations of continuously high prices for energy crops [54–56]. Promoting renewable energy use by law seems thus to counteract the aim of EU agricultural policy to diversify the agricultural landscape [54]. Crop market prices were recently decoupled from subsidies in the EU in favour of self-regulating agricultural diversification following portfolio theory [13–15]. With the relatively stable, high price level for energy crops, achieving a portfolio effect by portfolio diversification was thus a less attractive means to secure revenue for Bavarian farmers. New amendments already aim to reduce the strong impact of the EEG on agricultural market situations, but the consequences of the EEG will last into the future [55]. Thus, nowadays, relying on decoupled market situations is not enough to guarantee diversification of the agricultural landscape in the study area.

## Conclusion

We show that composition of crop portfolios is related to socio-economic farm characteristics and constrained by local soil quality and farm size. Furthermore, our results suggest that more diverse crop portfolios currently do not promote a higher revenue stability from arable land use in the study area, where policy still indirectly influences market prices of energy crops. Thus, at present, revenue stability does not motivate diversification of crop portfolios.

Diversification in agricultural landscapes, however, is important to maintain provisioning and non-provisioning ecosystem services that benefit farmers as well as the public. Indeed, our results show that especially small farms did not maintain high portfolio diversity. Especially in Bavaria, with its many small farms, maintenance of ecosystem services by on-site diversification might be difficult to realize at farm level. Therefore, appropriate diversification schemes could be regionally adjusted among farms because the economic value of ecosystem services maintained by diversified land use is expressed at the landscape scale. Accordingly, EU’s rural development and environmental protection policy should aim at increasing crop diversity on the regional scale across conglomerates of smaller farms. Since those smaller farms naturally maintain agrobiodiversity on the local scale due to their smaller field size, homogenization of arable land use should in turn be tolerated at individual farms with smaller fields. The strong impact of location and farm characteristics on portfolio composition shown in this study suggests that respective policy schemes should be adapted to farm types at the local scale.

## Supporting information

S1 Fig Spatial temperature and precipitation patterns in Bavaria based on the period 1950–2000. Annual temperature range is given as difference of maximum and minimum monthly temperature. Precipitation seasonality is the coefficient of variance of the monthly precipitation sums. Each scale is divided into percentiles of 10%. Data source: [57].
(PDF)

S2 Fig Overall silhouette width. Overall silhouette width was used as a criterion to detect the optimal number *k* of portfolios. First, clustering was applied with smaller sample size *s* and larger number of samples *S* for *k* = (2, …, 65) (black line) and consequently with larger sample size *s* and smaller number of samples *S* for *k* = 20, …, 30 (grey line).
(PDF)

S3 Fig Error of prediction of random forest model predicting classes of empirical crop portfolios based on clara clusters. Out of bag error (the prediction error for classifying each data sub-set that was not part of boot-strapped sub-samples) of random forest model predicting empirical crop portfolios based on clara clusters. The error is reported as out of bag error of the whole model and of each predicted portfolio.
(PDF)

S4 Fig Spatial distribution and frequency (height of bars) of the three most important empirical crop portfolios in urban district. Labels are inherited from the most dominant crops (accounting for more than 50% of the area of each portfolio).
(PDF)

S5 Fig Acreage (size of arable land) of empirical crop portfolios. Boxes refer to the first quartile, median, and third quartile of the data. Colours refer to grain dominated portfolios (red), maize dominated portfolios (green), portfolios being dominated by grain, maize, and other crops (purple) and other portfolios (grey).
(PDF)

S6 Fig Time series of yield for single crops that were used in empirical crop portfolios in Bavaria 2000–2013. (PDF)

S7 Fig Time series of revenue for empirical crop portfolios 2000–2013. Portfolios are labelled according to their most dominant crops. Spring barley and winter barley are abbreviated with *barley s* and *barley w*.
(PDF)

S1 Table Land use shares and occurrence of crops in the entire study area. Land use shares and occurrence of crops in the entire study area. Area shares represent the percentage of the total area of arable land in Bavaria with *A* = 2 052 177 ha. Occurrence represents the number of farms cultivating each crop. Number of observed farms was *N* = 79 532. Each farm cultivated one or several crops.
(PDF)

S2 Table Crops and crop categories which described land use of agricultural enterprises sampled for the agricultural survey lz2010. Some crops also included related crops, which were only marginally present in Bavaria: Wheat also included spelt and einkorn wheat; Rye included winter mixed grain. In some cases, the classification involved future use, which is indicated by superscript letters.
(PDF)

S3 Table Socio-economic variables. Socio-economic variables (categorical and numerical) were available to describe location, whole farm revenue, type of farming, and size of arable land of the agricultural enterprises sampled for the *LZ2010*. Organic farming refers to EU Regulation for Organic Food Farming EC No 834/2007. Administrative regions in Bavaria refer to NUTS 2 Regions of the EU.
(PDF)

S4 Table Training set selection, parameter setting, and quality of applied random forest analyses. Training set selection, parameter setting, and quality of applied random forest analyses. The *n*_*tree*_ bootstrapped sub-samples in random forest analysis were drawn in a quasi-stratified mode. Classes to be predicted were defined as strata. From each strata class a maximum of 40 objects (or less for smaller classes) was drawn for each bootstrap sub-sample. *n*_*tree*_ is the amount of decision trees grown, *m* the amount of predictors considered at each within-tree split. OOB is the out of bag error rate referring to the training set.
(PDF)

S5 Table Land use of portfolios based on cluster analysis (clara). Each portfolio was labeled according to its most important crops (contributing in sum to ≥ 50% of the area). The values in the table cells refer to the share of area [%] of each crop within each portfolio. The number of farms that cultivated each portfolio is given as *N*. *S* is the Sharpe-ratio and *H*′ the Shannon–Wiener diversity.
(PDF)

S1 Appendix List of R packages. (PDF)
