# Supplementary material for: Crop diversity and stability of revenue on farms in Central Europe: An analysis of big data from a comprehensive agricultural census in Bavaria
Source: PLoS One. 2018 Nov 19;13(11):e0207454. doi: 10.1371/journal.pone.0207454 (PMC6242357; doi:10.1371/journal.pone.0207454)
Supplement: S1 Fig — Annual temperature range is given as difference of maximum and minimum monthly temperature. Precipitation seasonality is the coefficient of variance of the monthly precipitation sums. Each scale is divided into percentiles of 10%. Data source: [57]. (PDF) [file pone.0207454.s001.pdf]

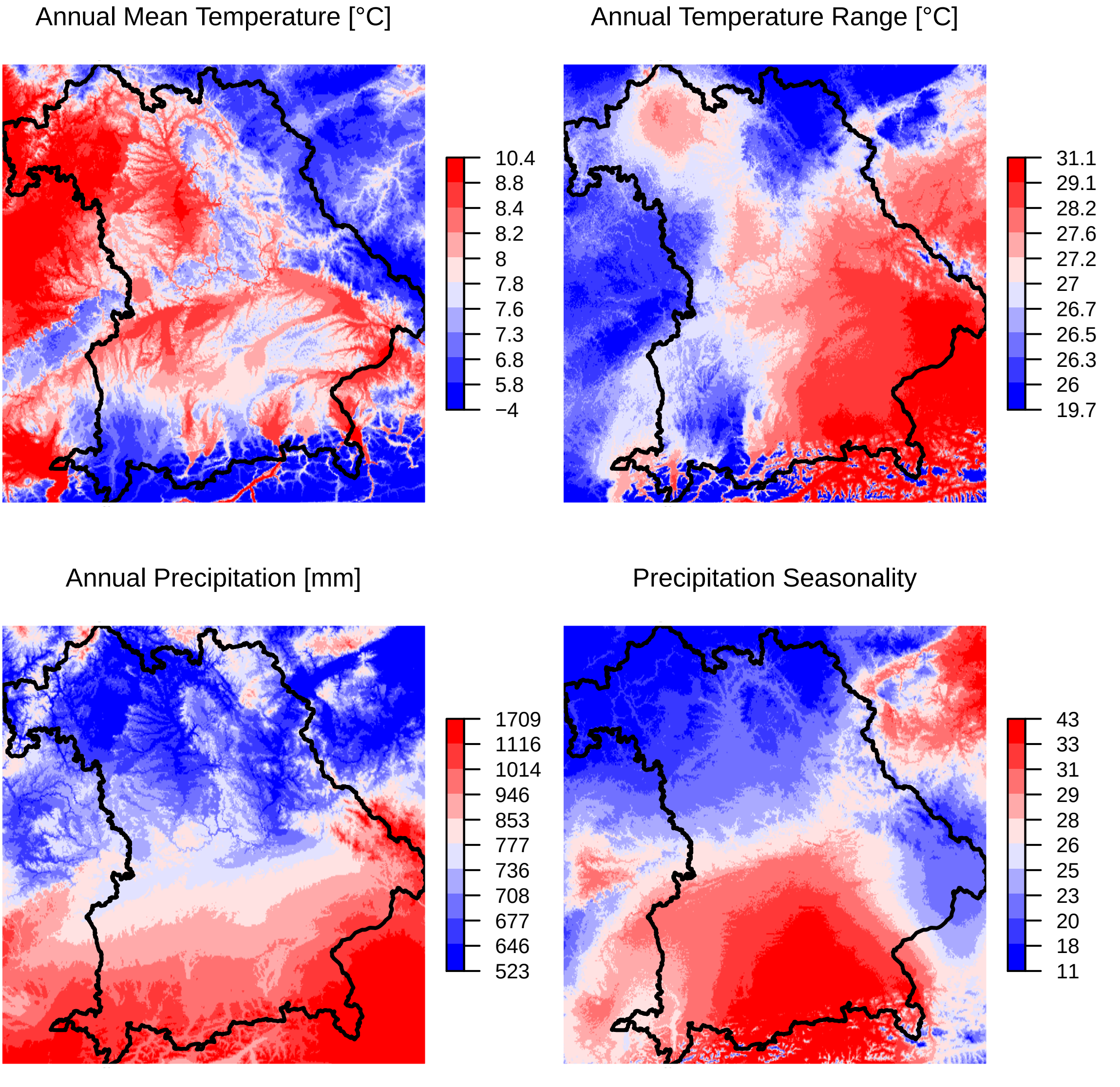
Annual Mean Temperature [°C]
10.4
8.8
8.4
8.2
8
7.8
7.6
7.3
6.8
5.8
−4
Annual Temperature Range [°C]
31.1
29.1
28.2
27.6
27.2
27
26.7
26.5
26.3
26
19.7
Annual Precipitation [mm]
1709
1116
1014
946
853
777
736
708
677
646
523
Precipitation Seasonality
43
33
31
29
28
26
25
23
20
18
11
