# Supplementary figures and images for: Crop diversity and stability of revenue on farms in Central Europe: An analysis of big data from a comprehensive agricultural census in Bavaria
Source: PLoS One. 2018 Nov 19;13(11):e0207454. doi: 10.1371/journal.pone.0207454 (PMC6242357; doi:10.1371/journal.pone.0207454)

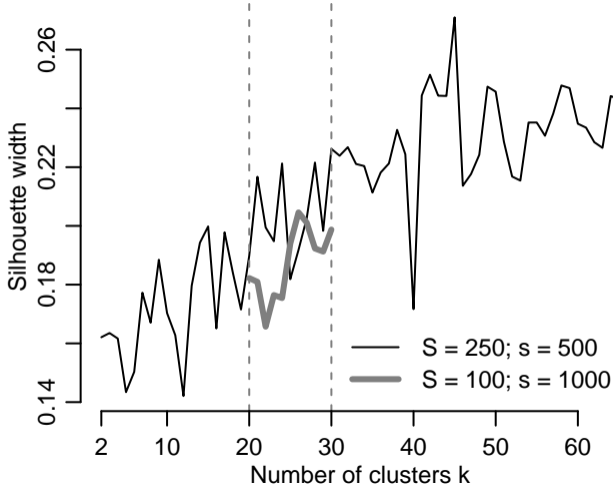

Supplement: S2 Fig — Overall silhouette width was used as a criterion to detect the optimal number k of portfolios. First, clustering was applied with smaller sample size s and larger number of samples S for k = (2, …, 65) (black line) and consequently with larger sample size s and smaller number of samples S for k = 20, …, 30 (grey line). (PDF) [file pone.0207454.s002.pdf]

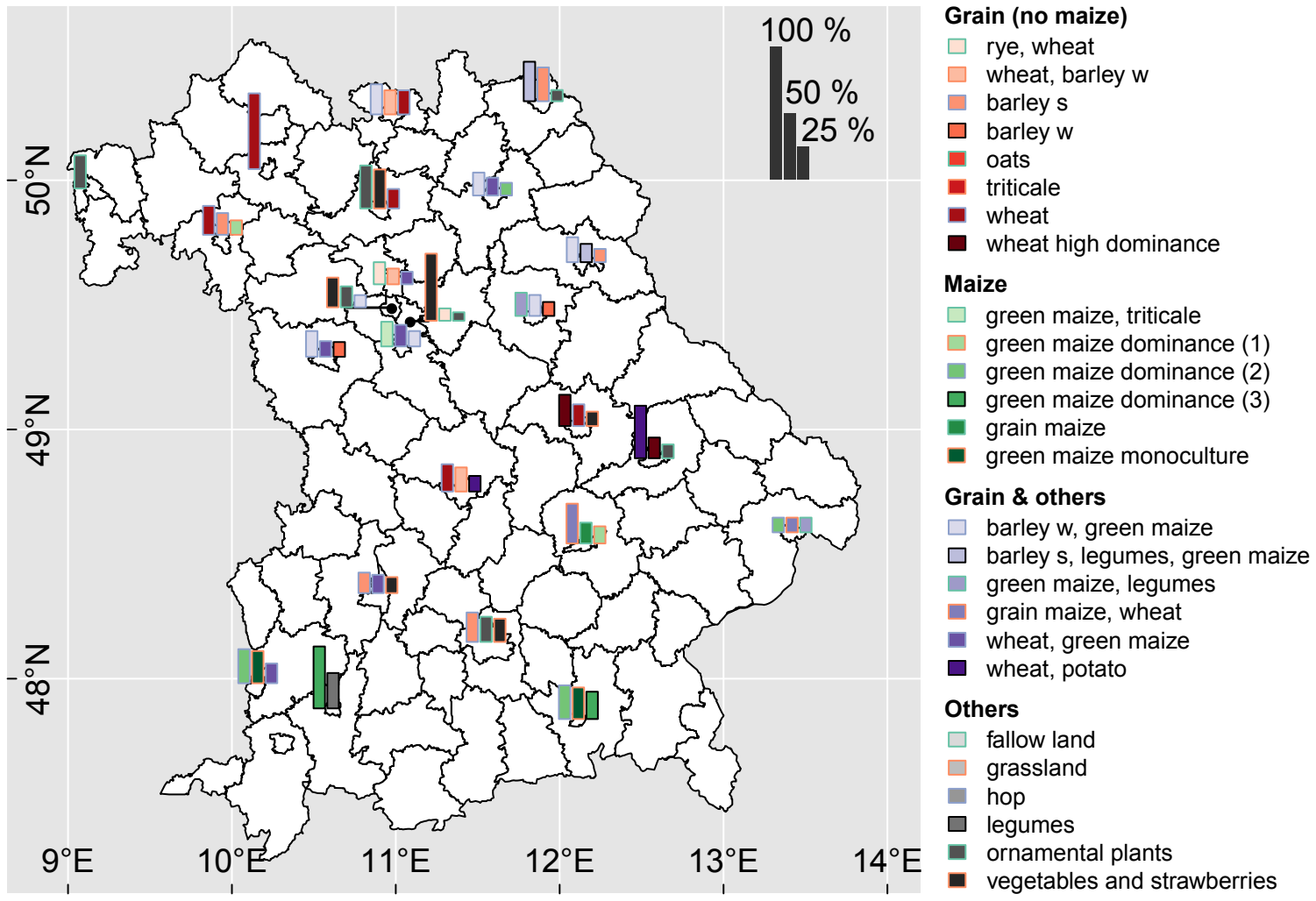

Supplement: S4 Fig — Labels are inherited from the most dominant crops (accounting for more than 50% of the area of each portfolio). (PDF) [file pone.0207454.s004.pdf]

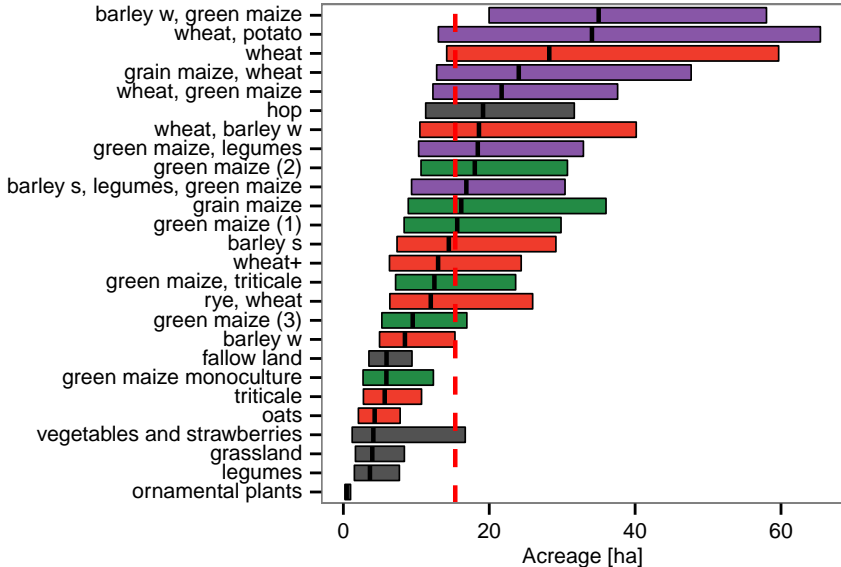

Supplement: S5 Fig — Boxes refer to the first quartile, median, and third quartile of the data. Colours refer to grain dominated portfolios (red), maize dominated portfolios (green), portfolios being dominated by grain, maize, and other crops (purple) and other portfolios (grey). (PDF) [file pone.0207454.s005.pdf]

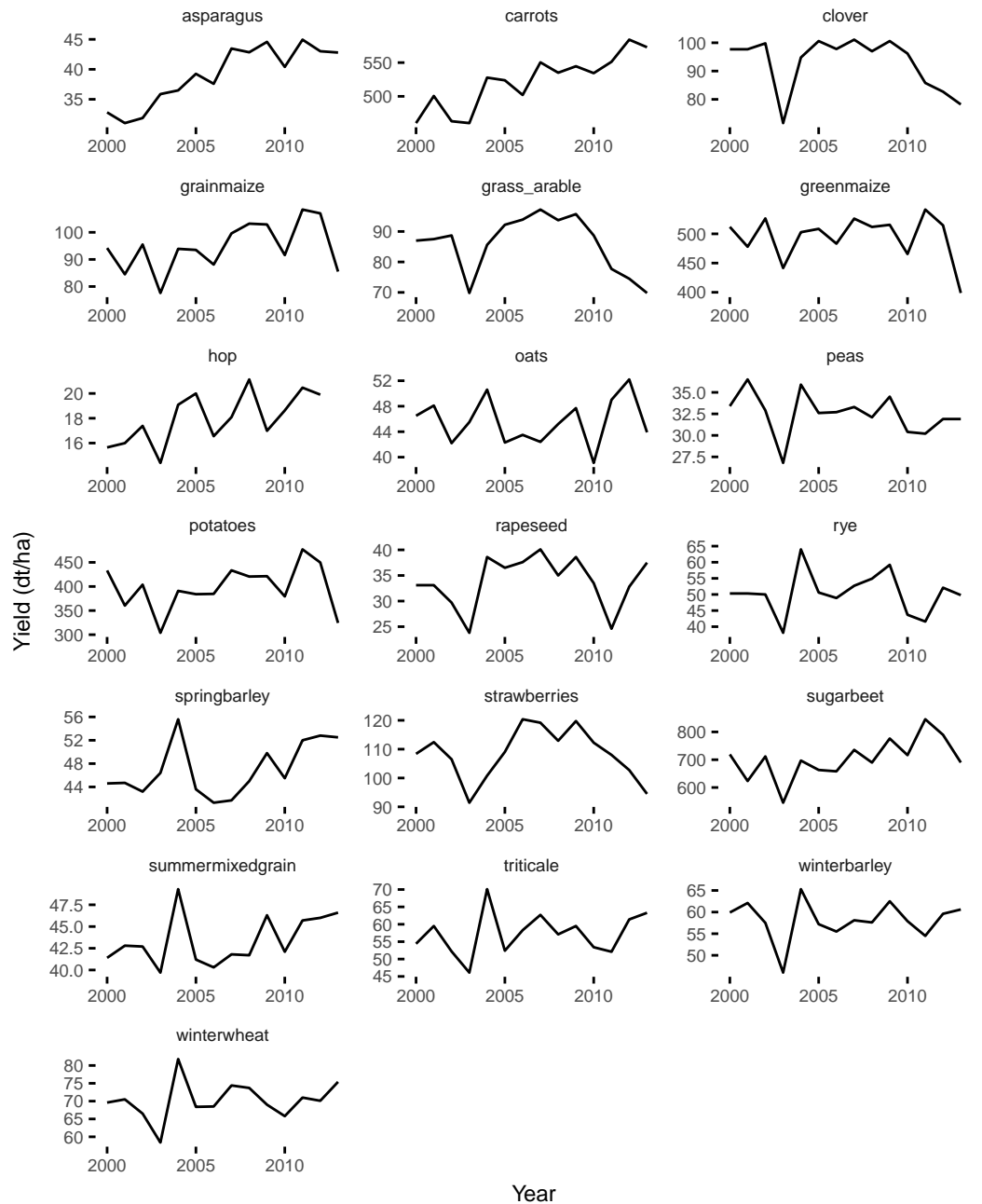

Supplement: S6 Fig — (PDF) [file pone.0207454.s006.pdf]

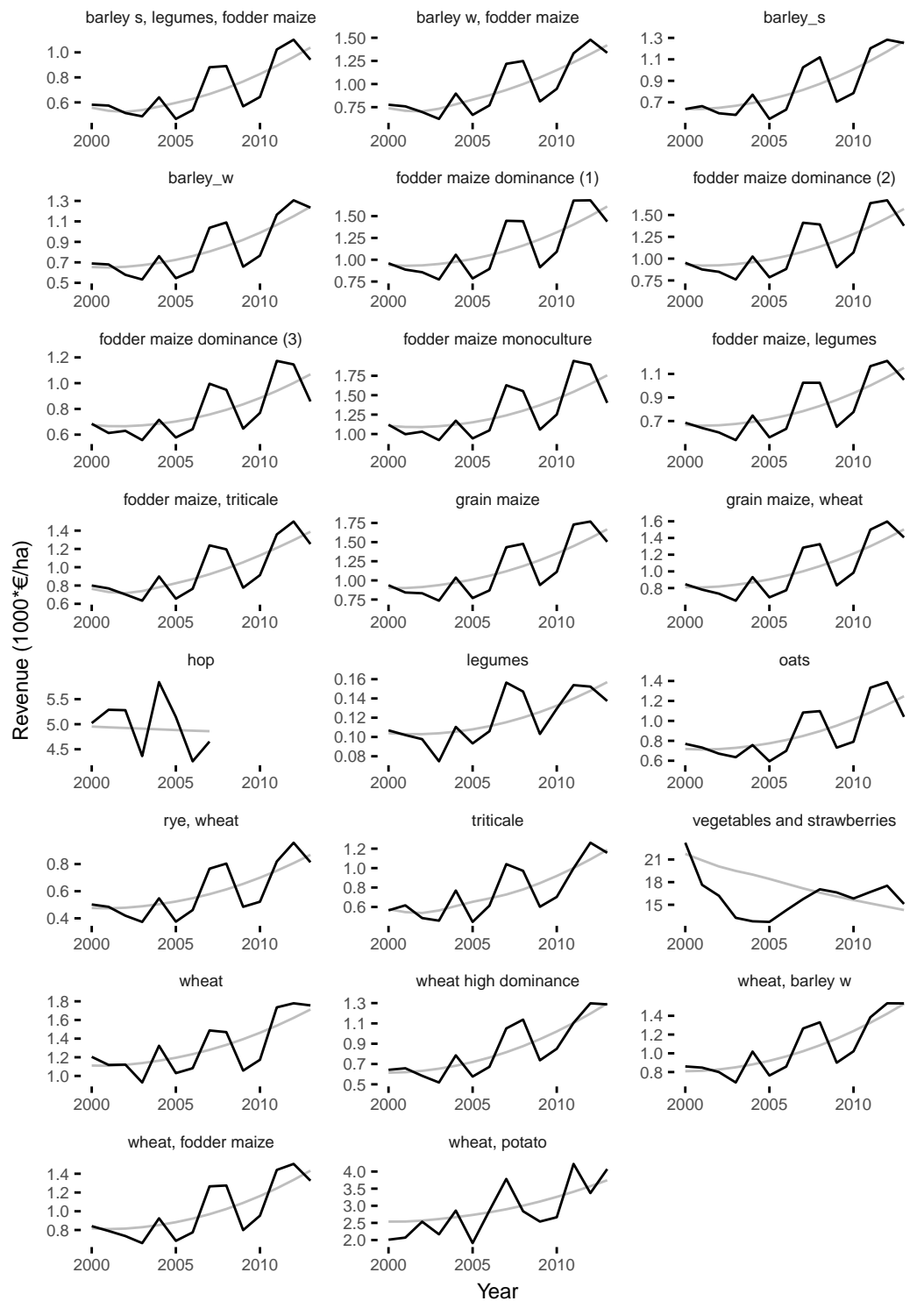

Supplement: S7 Fig — Portfolios are labelled according to their most dominant crops. Spring barley and winter barley are abbreviated with barley s and barley w. (PDF) [file pone.0207454.s007.pdf]
