# Supplementary material for: Crop diversity and stability of revenue on farms in Central Europe: An analysis of big data from a comprehensive agricultural census in Bavaria
Source: PLoS One. 2018 Nov 19;13(11):e0207454. doi: 10.1371/journal.pone.0207454 (PMC6242357; doi:10.1371/journal.pone.0207454)
Supplement: S3 Fig — Out of bag error (the prediction error for classifying each data sub-set that was not part of boot-strapped sub-samples) of random forest model predicting empirical crop portfolios based on clara clusters. The error is reported as out of bag error of the whole model and of each predicted portfolio. (PDF) [file pone.0207454.s003.pdf]

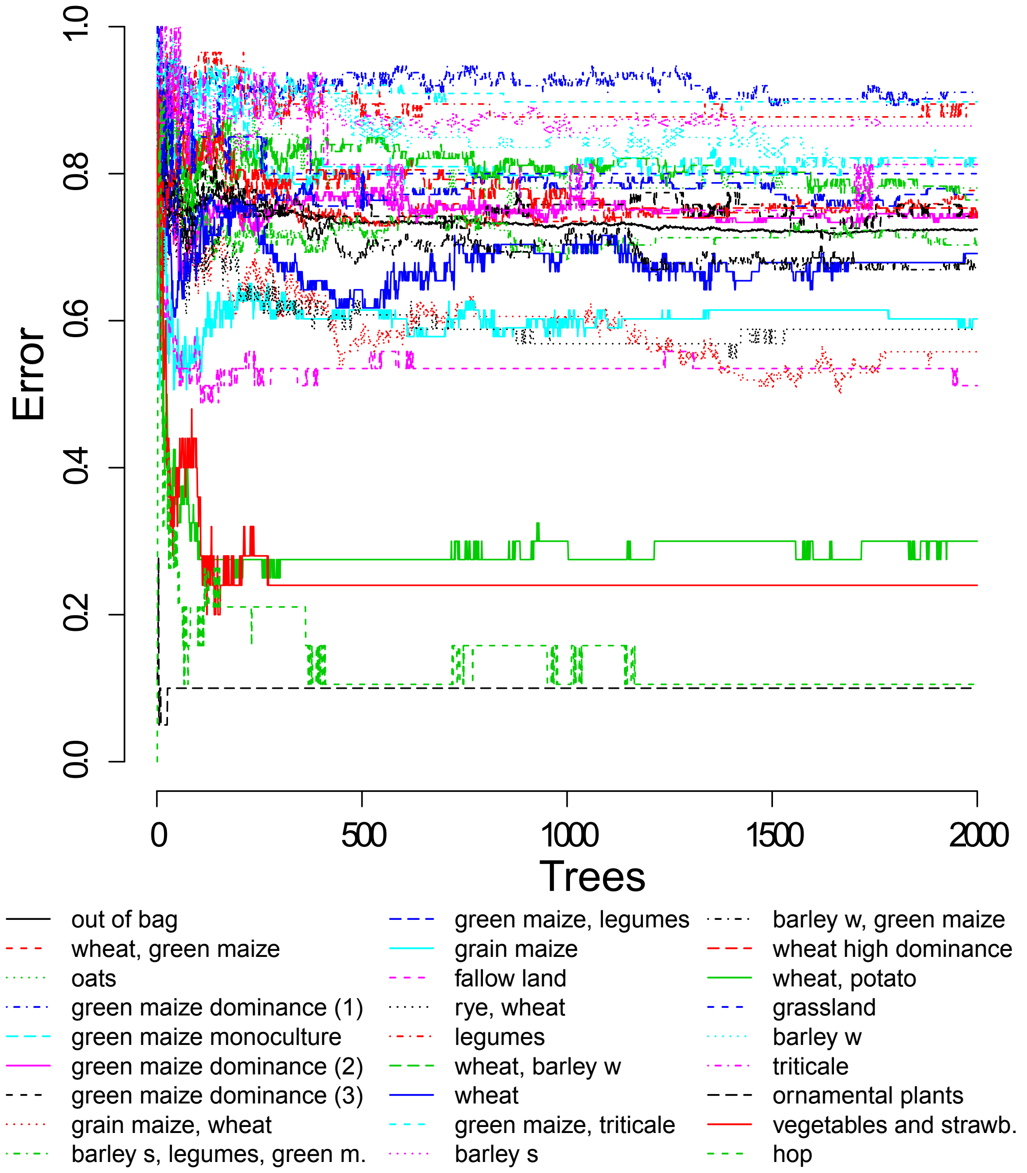
Error
0.0
0.2
0.4
0.6
0.8
1.0
0
500
1000
1500
2000
Trees
out of bag
wheat, green maize
oats
green maize dominance (1)
green maize monoculture
green maize dominance (2)
green maize dominance (3)
grain maize, wheat
barley s, legumes, green m.
green maize, legumes
grain maize
fallow land
rye, wheat
legumes
wheat, barley w
wheat
green maize, triticale
barley s
barley w, green maize
wheat high dominance
wheat, potato
grassland
barley w
triticale
ornamental plants
vegetables and strawb.
hop
