# Supplementary material for: Crop diversity and stability of revenue on farms in Central Europe: An analysis of big data from a comprehensive agricultural census in Bavaria
Source: PLoS One. 2018 Nov 19;13(11):e0207454. doi: 10.1371/journal.pone.0207454 (PMC6242357; doi:10.1371/journal.pone.0207454)
Supplement: S1 Table — Land use shares and occurrence of crops in the entire study area. Area shares represent the percentage of the total area of arable land in Bavaria with A = 2 052 177 ha. Occurrence represents the number of farms cultivating each crop. Number of observed farms was N = 79 532. Each farm cultivated one or several crops. (PDF) [file pone.0207454.s008.pdf]

Crop	Area share [%]	Occurrence
Winter wheat	24.7	55122
Fodder maize	18.3	44308
Winter barley	14.1	44425
Winter rapeseed	6.7	18944
Spring barley	5.5	18204
Grain maize	5.3	12869
Legumes (whole crop harvest)	5.1	29800
Triticale	3.6	16789
fallow land	2.7	20138
Oats	2.4	16438
Potatoes	2.2	14781
Sugar beet	2.1	9985
Rye	2.0	7687
Grassland on arable land (Field grass)	1.0	10551
Vegetables and strawberries	0.77	3904
Hop	0.77	1242
Peas	0.58	4511
Mixed grain (fodder, dough-ripe)	0.51	2318
Spring wheat	0.35	2159
Summer mixed grain	0.32	1746
Further mixed crops for whole plant harvest	0.15	884
Field bean	0.14	853
Mustard, poppy, soy (grain)	0.13	1104
Further energy crops	0.12	623
Unused land	0.10	4906
Sunflowers	< 0.10	415
Agricultural seed production		325
Beets, turnips, carrots (fodder)		1686
Ornamental plants		1162
Medical/aromatic/spice plants		198
Durum		196
Millet, Sorghum, Canary seed		164
Tobacco		18
Further pulses (grain harvest)		156
Sweet lupine		158
Spring rapeseed		80
Horticultural seed production		NA
Lineseed		NA
Hemp		NA
Flax, kenaf ao (fibre)		NA
Further industrial plants		364
Others		73
