# Supplementary material for: Crop diversity and stability of revenue on farms in Central Europe: An analysis of big data from a comprehensive agricultural census in Bavaria
Source: PLoS One. 2018 Nov 19;13(11):e0207454. doi: 10.1371/journal.pone.0207454 (PMC6242357; doi:10.1371/journal.pone.0207454)
Supplement: S2 Table — Some crops also included related crops, which were only marginally present in Bavaria: Wheat also included spelt and einkorn wheat; Rye included winter mixed grain. In some cases, the classification involved future use, which is indicated by superscript letters. (PDF) [file pone.0207454.s009.pdf]

| Groups of crops | Crops and crop categories |
|---|---|
| Grain[G,S] | Winter wheat ao |
| | Spring wheat |
| | Durum |
| | Rye ao |
| | Triticale |
| | Winter barley |
| | Spring barley |
| | Oats |
| | Summer mixed grain |
| | Grain maize |
| | Millet, sorghum, canary seed ao |
| Pulses[G,S] | Peas |
| | Field bean |
| | Sweet lupine |
| | Further pulses (grain harvest) |
| Horticultural products | Vegetables and strawberries |
| | Ornamental plants |
| | Horticultural seed production |
| | Agricultural seed production |
| | Others |
| Further industrial crops[I] | Hop |
| | Tobacco |
| | Medical/aromatic/spice plants |
| | Hemp |
| | Flax, kenaf ao (fibre) |
| | Further crops for energy production only |
| | Further industrial plants |
| Oil seeds[G,S] | Winter rapeseed |
| | Spring rapeseed |
| | Sunflowers |
| | Linseed |
| | Mustard, poppy, soy ao |
| Root crops | Potato[F,G,I,S] |
| | Sugar beet[E,G] |
| | Beets, turnips, carrots [F,G] |
| Mixed grain[incl. G] | Mixed grain[incl. G] |
| | Fodder maize |
| | Legumes (whole crop harvest) |
| | Grassland on arable land |
| | Further mixed crops for whole plant harvest |
| Fallow land | Fallow land |

E: use as energy crop, F: fodder production, G: grain/food production, I: industrial use , S: seed production
