# Supplementary material for: Crop diversity and stability of revenue on farms in Central Europe: An analysis of big data from a comprehensive agricultural census in Bavaria
Source: PLoS One. 2018 Nov 19;13(11):e0207454. doi: 10.1371/journal.pone.0207454 (PMC6242357; doi:10.1371/journal.pone.0207454)
Supplement: S3 Table — Socio-economic variables (categorical and numerical) were available to describe location, whole farm revenue, type of farming, and size of arable land of the agricultural enterprises sampled for the LZ2010. Organic farming refers to EU Regulation for Organic Food Farming EC No 834/2007. Administrative regions in Bavaria refer to NUTS 2 Regions of the EU. (PDF) [file pone.0207454.s010.pdf]

Group	Variable	Class/unit
Categorical	Organic farming	Yes/No
	Type of farming	Arable farming<br>Gardening<br>Permanent crop<br>Fodder<br>Husbandry<br>Diverse crop farming<br>Livestock<br>Diverse farming<br>Unclassified
	Economical type	Full time farming<br>Part time farming
	Administrative district	Lower Bavaria<br>Upper Bavaria<br>Lower Franconia<br>Middle Franconia<br>Upper Franconia<br>Upper Palatinate<br>Swabia
Numerical	Whole farm revenue	[€]
	Acreage	[ha]
	Inter cropping, winter	[%]
	Inter cropping, summer	[%]
