# Supplementary material for: Crop diversity and stability of revenue on farms in Central Europe: An analysis of big data from a comprehensive agricultural census in Bavaria
Source: PLoS One. 2018 Nov 19;13(11):e0207454. doi: 10.1371/journal.pone.0207454 (PMC6242357; doi:10.1371/journal.pone.0207454)
Supplement: S4 Table — Training set selection, parameter setting, and quality of applied random forest analyses. The ntree bootstrapped sub-samples in random forest analysis were drawn in a quasi-stratified mode. Classes to be predicted were defined as strata. From each strata class a maximum of 40 objects (or less for smaller classes) was drawn for each bootstrap sub-sample. ntree is the amount of decision trees grown, m the amount of predictors considered at each within-tree split. OOB is the out of bag error rate referring to the training set. (PDF) [file pone.0207454.s011.pdf]

	Training set	Stratum size	n_{tree}	m	OOB error (training)/ classification error (test)
Predicting empirical crop portfolios from *CLARA* analysis	Best sub-sample from *CLARA* analysis; $N_{train} = 2000$	$16 < n_h < 40$	2000	3	72.4 %/74.0 %
