# Supplementary material for: Crop diversity and stability of revenue on farms in Central Europe: An analysis of big data from a comprehensive agricultural census in Bavaria
Source: PLoS One. 2018 Nov 19;13(11):e0207454. doi: 10.1371/journal.pone.0207454 (PMC6242357; doi:10.1371/journal.pone.0207454)
Supplement: S5 Table — Each portfolio was labeled according to its most important crops (contributing in sum to ≥ 50% of the area). The values in the table cells refer to the share of area [%] of each crop within each portfolio. The number of farms that cultivated each portfolio is given as N. S is the Sharpe-ratio and H′ the Shannon–Wiener diversity. (PDF) [file pone.0207454.s012.pdf]

Portfolio label	Winter wheat	Rye	Triticale	Winter barley	Spring barley	Oats	Grain maize	Green maize	Legumes	Field grass	Sugar beet	Rapeseed	Hop	Fallow land	Vegetables*	Ornamental plants	Potatoes	S	H'	N
ornamentals																100.0		NA	0.0	602
vegetables															100.0			10.8	1.1	705
hop													89.6	10.4				10.4	0.3	719
grassland										100.0								NA	0.0	644
legumes									100.0									6.8	0.0	2060
fallow land														100.0				NA	0.0	1987
GM mono								100.0										5.3	0.0	3297
GM (1)	48.2							51.8										5.3	0.7	4825
GM (2)	24.4			19.5				56.1										5.4	1.0	6314
GM (3)								57.0	43.0									5.4	0.7	2531
GM, triticale			34.3	29.4				36.3										5.3	1.1	3641
grain maize	42.2						57.8											5.2	0.7	2872
GM, legumes	26			19.0				28.9	25.9					0.1				5.4	1.4	5183
wheat, GM	38.4			25.1				32.6	3.9									5.3	1.2	6935
SB, legumes, GM			14.5	15.7	25.0	8.9		16.3	19.6									5.2	1.7	4219
WB, GM	22		9.3	28.4				23.6				15.9		0.8				5.7	1.6	4743
grain maize, wheat	35.2			29.5			35.3											5.2	1.1	3776
wheat, potato	46.8										17.7						35.6	5.8	1.0	1863
oats						61.9		38.1										5.0	0.7	1229
WB	30.7			50.6	13.7	5.0												5.1	1.1	2833
triticale			100.0															4.5	0.0	693
rye, wheat	21.9	37.9				18.7			21.6									4.7	1.3	2294
wheat+	77.7										22.3							5.8	0.5	2726
wheat, WB	32.2			32.7								28.4		6.7				6.5	1.3	4677
SB	24.9			5.5	52.6							16.7					0.3	5.4	1.2	4560
wheat	53.8			14.1							7.3	24.8						7.1	1.1	3604

GM: green maize, SB: spring barley, WB: winter barley

*) The crop category *vegetables and strawberries*, here called *vegetables*, was assumed to consist of 1/3 asparagus, 1/3 carrots, and 1/3 strawberries. Thus, S for the portfolio *vegetables and strawberries* was calculated from the price and yield data of those three crops. H' was calculated analogously.
