# Supplementary material for: Crop diversity and stability of revenue on farms in Central Europe: An analysis of big data from a comprehensive agricultural census in Bavaria
Source: PLoS One. 2018 Nov 19;13(11):e0207454. doi: 10.1371/journal.pone.0207454 (PMC6242357; doi:10.1371/journal.pone.0207454)
Supplement: S1 Appendix — (PDF) [file pone.0207454.s013.pdf]

List of R packages

The calculations were done using packages CLUSTER (Maechler et al., 2014), RANDOMFOREST (Liaw and Wiener, 2002), and PSYCH (Revelle, 2016) in R (R Core Team, 2014). For mapping and plotting we used packages RASTER (Hijmans, 2015), GGPLOT2 (Wickham, 2009) and RWORLDXTRA (South, 2012).

References

Hijmans, Robert J. (2015). *raster: Geographic Data Analysis and Modeling*. R package version 2.3-40. URL: `http://CRAN.R-project.org/package=raster`.

Liaw, Andy and Matthew Wiener (2002). "Classification and regression by randomForest". In: *R News* 2.3, pp. 18–22. URL: `http://cran.r-project.org/doc/Rnews/`.

Maechler, Martin et al. (2014). *cluster: cluster analysis basics and extensions*.

R Core Team (2014). *R: A language and environment for statistical computing*. Vienna, Austria.

Revelle, William (2016). *psych: Procedures for Psychological, Psychometric, and Personality Research*. R package version 1.6.4. Northwestern University. Evanston, Illinois. URL: `http://CRAN.R-project.org/package=psych`.

South, Andy (2012). *rworldxtra: Country boundaries at high resolution*. URL: `http://cran.r-project.org/package=rworldxtra`.

Wickham, Hadley (2009). *ggplot2: elegant graphics for data analysis*. Springer New York. ISBN: 978-0-387-98140-6. URL: `http://had.co.nz/ggplot2/book`.
